# Antagonism of Various Diseases

**Published:** 1844-01-13

**Authors:** 


### ANTAGONISM OF VARIOUS DISEASES.

*Comparative Infrequency of Consumption and Typhoid Fever in marshy Districts.*

BY M. BOUDIN.

M. Boudin has, we believe, succeeded in establishing the fact, that both phthisis and typhoid fever are extremely rare in marshy districts, that this infrequency depends on a kind of protective agency exerted by the marsh miasm, and that the immunity is always proportioned to the degree of *impaludation.* It had been shown by M. Chassinat that phthisis is much more prevalent among the galley-slaves at Toulon than at Rochefort; and that amongst the galley-slaves at Brest, also, the victims of that disease are nine times more numerous than at Rochefort, which is proverbially marshy. Having decidedly ascertained the influence of a marshy soil in the department of *Charente Inferieure,* M. Boudin wished to obtain certain data respecting the frequency of phthisis and typhoid fever in the marshy localities of *l'Ain.* For this purpose he wrote to several physicians, and especially to M. Nepple, from whose answer the following is an extract:—"For my part," says M. Nepple, "I have not the slightest doubt of the scarcity of phthisis in very marshy places, and this scarcity has always appeared to me to be in direct relation with the intensity of *impaludation.* Thus, whilst in the *communes,* situated in the midst of the marshy country, there does not occur a single case of phthisis, we find the number of them constantly increasing in proportion as we recede from the marshes. So that, at a certain limit, we find tubercles and intermittent fevers co-existing; but under these circumstances, the endemic intermittent is but of little intensity.

"Thus, at Montreuil, phthisis is any thing but rare, though intermittent fever occurs annually, but the miasmata producing this fever, before they can reach the town, have to pass the distance of a quarter of a league, and their influence is slight, superficial, temporary, and purely productive of fever. The entire system is not influenced or modified in such a durable way by them as to oppose the development of tubercle. It is altogether different in the midst of the marshy districts."

The following is an extract of a letter from M. Picoud, of Bourg, on the same subject:—"After more than forty-five years of practice, I have never found a case in opposition to your observations. I have in vain taxed my memory and consulted my notes; I have not met with any trace of tubercular disease occurring in the marshy districts. Wishing seriously to come to a true decision, I have not depended on myself alone, but have consulted many of my colleagues, and especially Dr. Huldelet, who has been for a long time physician to the hospital, and who has an extensive practice in the country about Villard, Malieux, and other *communes* situated in the centre of the marshes; and he cannot bring to mind a single instance of phthisis occurring in those districts. I have remarked that the children of wealthy parents, who are sent from home to be educated, lose the benefit of the marshy country."—*Provincial Journal, from Gaz. des Hopitaux,* September 2, 1843.

